# Timed Barium Esophagography in Esophagogastric Junction Outflow Obstruction: A Retrospective Radiologic Assessment

**DOI:** 10.3390/diagnostics16172743

**Published:** 2026-08-27

**Authors:** Vittorio Patanè, Piera Senneca, Teresa Giuffrè, Marta Pagliaro, Antonio Cefaliello, Matteo Flaminio, Mario Ricchiuti, Anna Russo, Maria Chiara Brunese, Giovanni Sarnelli, Roberto Grassi, Marcella Pesce, Alfonso Reginelli

**Affiliations:** 1 Precision Medicine Department, University of Campania “Luigi Vanvitelli”, Piazza Luigi Miraglia 2, 80138 Naples, Italy; 2 Gastroenterology Unit, Hospital “San Giuseppe Moscati”, 81031 Aversa, Italy; 3 Gastroenetology Unit, Pineta Grande Hospital, 81030 Castel Volturno, Italy; 4 Department of Clinical Medicine and Surgery, University of Naples Federico II, 80131 Naples, Italy

**Keywords:** timed barium esophagography, esophagogastric junction outflow obstruction, EGJOO, achalasia, esophageal motility disorders, functional imaging, gastrointestinal radiology

## Abstract

**Introduction:** Esophagogastric junction outflow obstruction (EGJOO) is a heterogeneous manometric pattern requiring symptoms and supportive evidence to establish clinical relevance. Timed barium esophagography (TBE) provides functional radiologic assessment of esophageal emptying through timed imaging, quantitative barium column measurements, and morphologic evaluation. **Methods:** This single-center retrospective study included 42 patients referred for TBE for suspected EGJOO between June 2024 and December 2025. TBE was performed upright after ingestion of low-density barium suspension. Positivity required a continuous residual esophageal barium column with craniocaudal height ≥ 2 mm at 1′ after bolus ingestion. Minimal mucosal coating, non-columnar traces, and contrast confined to a hiatal hernia were recorded qualitatively but not considered positive. Quantitative measurements, clearance patterns, morphology, interobserver reliability, and exploratory concordance with Chicago Classification v4.0-adjudicated categories were assessed. **Results:** TBE was positive in 12/42 patients (28.6%). Mean age was 58.0 ± 13.9 years; 26/42 patients were female. In positive studies, mean column height and width were 122.9 ± 65.4 mm and 31.1 ± 17.8 mm at 1 min, and 89.0 ± 54.6 mm and 28.2 ± 18.2 mm at 2 min. Persistent 5 min retention occurred in two patients. Interobserver reliability was excellent (ICC 0.90). Morphologic abnormalities were frequent in positive examinations, supporting overall radiologic phenotype-based interpretation. In the EGJOO-focused subset, exploratory concordance metrics were 41.7%, 100%, 100%, and 46.2% for sensitivity-like, specificity-like, positive predictive, and negative predictive concordance, respectively. **Conclusions:** In this small exploratory cohort, standardized TBE provided functional and morphologic information on esophageal bolus transit. Positive TBE findings showed exploratory concordance with clinically relevant EGJOO, whereas a negative study did not exclude it. These findings require validation in larger prospective cohorts.

## 1. Introduction

Esophagogastric junction outflow obstruction (EGJOO) is a heterogeneous manometric pattern characterized by impaired relaxation of the esophagogastric junction (EGJ) with preserved or partially preserved esophageal peristalsis [1,2,3]. In clinical practice, however, EGJOO does not represent a single disease entity. It may reflect clinically relevant obstruction, early or variant achalasia, structural or post-treatment narrowing, opioid-related dysmotility, or an incidental manometric finding without significant impairment of bolus transit [4,5].

This heterogeneity explains why the Chicago Classification v4.0 emphasizes that manometric findings should be interpreted together with symptoms and supportive evidence from complementary tests [6]. From a radiologic perspective, the central question is not only whether EGJ relaxation pressure is elevated, but whether this pressure abnormality produces a visible functional consequence during bolus transit [7,8]. A patient may show manometric evidence of EGJ outflow impairment and still clear a liquid bolus effectively, whereas another patient may demonstrate clinically meaningful retention above the EGJ [2,9,10,11].

Timed barium esophagography (TBE) is well-suited to address this question because it directly depicts esophageal emptying under standardized upright conditions [12,13,14]. Unlike high-resolution manometry, which measures intraluminal pressure, TBE provides a functional and morphologic radiologic assessment by combining timed image acquisition, quantitative barium column measurements, temporal clearance analysis, and evaluation of distal esophageal and EGJ morphology [15,16,17]. This allows the radiologist to document not only the presence or absence of residual barium retention, but also the degree of emptying impairment and the associated imaging pattern [10,18].

The value of TBE is therefore both quantitative and qualitative. Barium column height and width provide objective measurements that can be compared across time points, between examinations, and in multidisciplinary discussions [13,14,18]. At the same time, qualitative findings such as distal tapering, esophageal dilation, tertiary or dyskinetic contractions, corkscrew-like morphology, air-fluid level, diverticular outpouching, hiatal hernia, or mucosal irregularity may suggest alternative or coexisting disorders [19,20]. A purely numeric approach may miss clinically meaningful morphologic patterns, whereas an exclusively qualitative report may lack reproducibility. A structured radiologic approach should integrate both components [21,22].

Previous studies have often considered TBE mainly as a supportive test for manometric diagnoses [23,24]. However, in patients referred for suspected EGJOO, a radiology-centered assessment may provide additional value by separating true esophageal retention from non-obstructive findings such as mucosal coating or contrast confined to a hiatal hernia, and by classifying examinations according to clearance pattern and morphology [25,26,27]. This distinction is clinically relevant because a positive TBE may support the functional relevance of an EGJOO pattern, whereas a negative examination does not necessarily exclude clinically relevant EGJOO [10,28,29,30].

The aim of this study was to evaluate standardized TBE as a functional and morphologic radiologic examination in patients referred for suspected EGJOO. We focused on quantitative barium column measurements, temporal clearance patterns, qualitative morphologic findings, interobserver reliability, and exploratory concordance with Chicago Classification v4.0-adjudicated diagnostic categories.

## 2. Materials and Methods

The study was conducted in accordance with the Declaration of Helsinki and was approved by the local ethics committee of the University Hospital of Campania “L. Vanvitelli” and AORN “Ospedale dei Colli”, Naples, Italy, under a dedicated study protocol (Prot. N. 36985/i/2023). Because of the retrospective nature of the study, the requirement for written informed consent was waived by the ethics committee.

### 2.1. Study Design and Population

**Figure 1:**
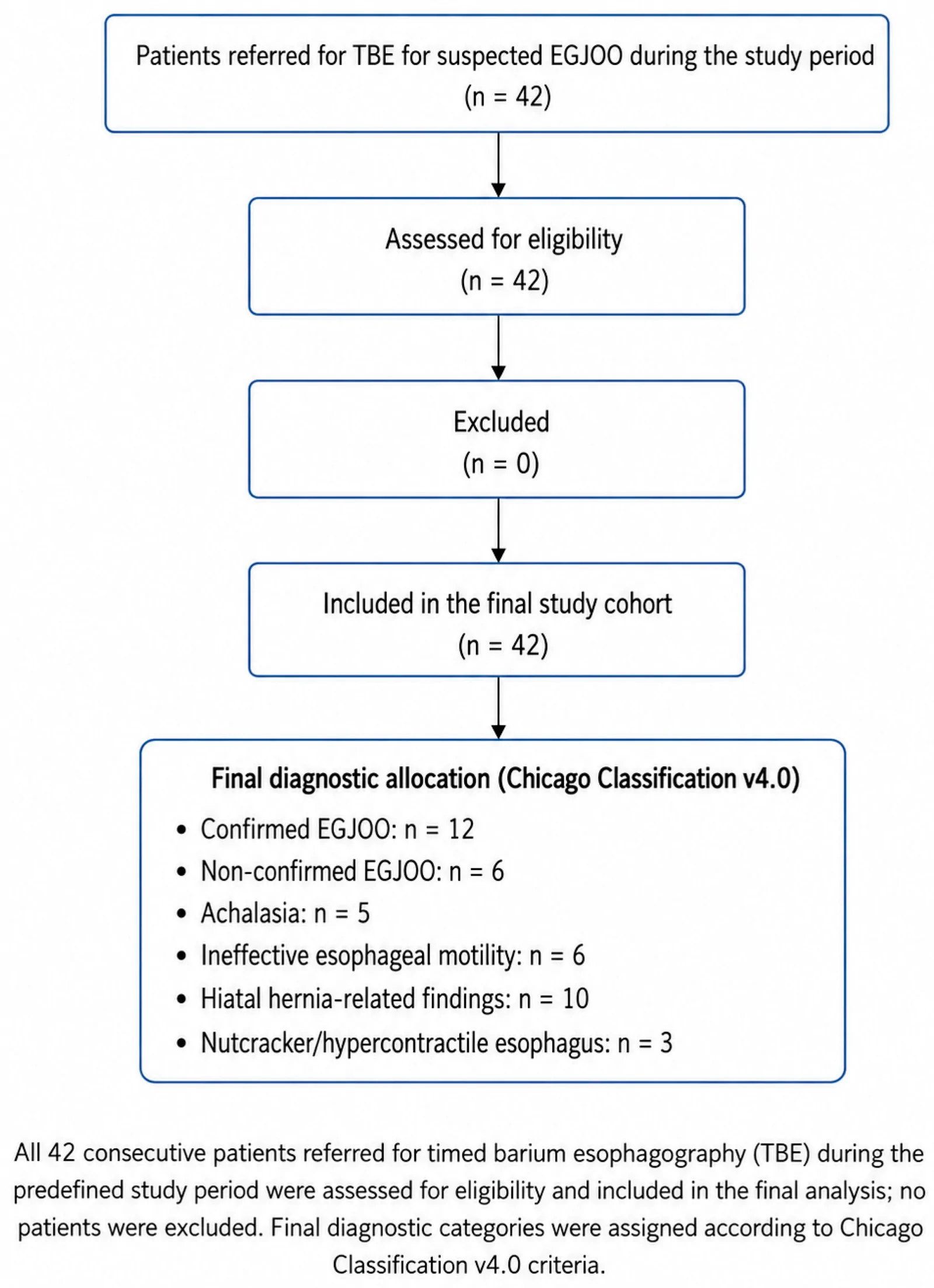
STROBE flow diagram of patient inclusion and final diagnostic allocation.

All 42 consecutive patients meeting the study eligibility criteria during the predefined study period were included in the final analysis, with no exclusions after screening. The patient selection process and final diagnostic allocation are summarized in Figure 1. All 42 consecutive patients meeting the study eligibility criteria during the predefined study period were included, with no exclusions after screening.

Patients were referred to the Radiology Department of the University Hospital “Luigi Vanvitelli” from the Gastroenterology Department of the University Hospital “Federico II”, both in Naples, Italy, between June 2024 and December 2025.

Before TBE referral, all patients underwent a stepwise clinical and instrumental assessment. Symptoms suggestive of an esophageal motility disorder were initially evaluated and quantified using the Eckardt score. All patients subsequently underwent high-resolution manometry (HRM), followed by TBE as a complementary functional imaging examination.

Patient demographics, TBE results, quantitative barium column measurements, qualitative radiologic descriptors, and high-resolution manometry-adjudicated diagnostic categories were extracted from the study database. The radiologists performing and interpreting TBE were blinded to the detailed clinical documentation and HRM findings at the time of image assessment. Final diagnostic categories were assigned according to Chicago Classification v4.0 criteria. Diagnostic classification was performed within the Chicago Classification v4.0 framework. In this study, ‘non-confirmed EGJOO’ referred to patients with a clinically and manometrically suspected EGJOO pattern in whom the diagnosis remained provisional because supportive evidence from ancillary testing had not yet established its clinical relevance. TBE was one of the ancillary examinations used within this diagnostic pathway.

### 2.2. Timed Barium Esophagogram Acquisition Protocol

**Figure 2:**
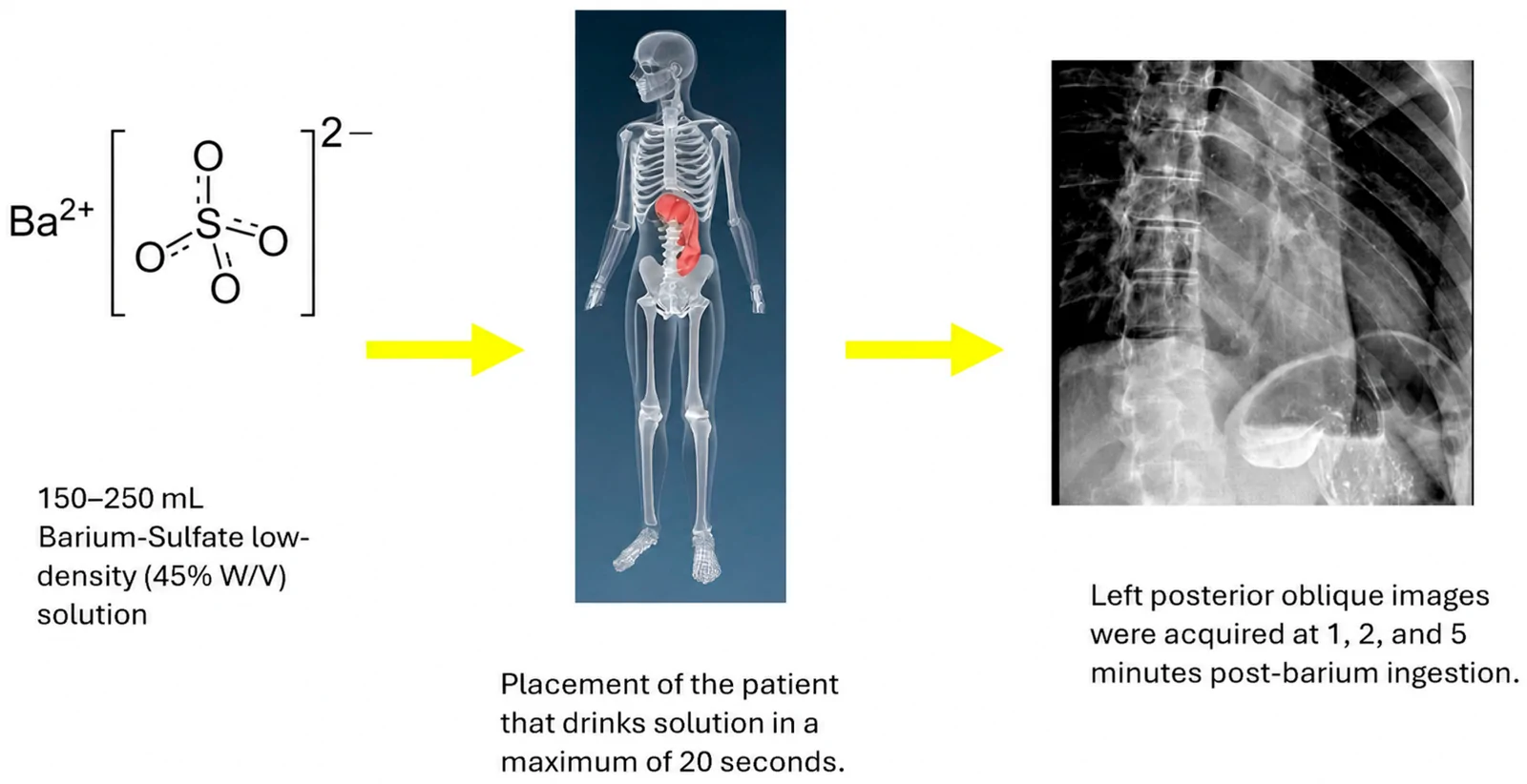
Schematic representation of the timed barium esophagogram protocol. Patients ingested 150–200 mL of low-density barium sulfate suspension, 45% weight/volume, within a standardized interval of up to 20 s. Spot radiographs were then acquired with the patient standing upright in the left posterior oblique projection at 1, 2, and, when indicated, 5 min after barium ingestion.

Radiographs were obtained using General Medica Merate Opera Swing device (GENERAL MEDICAL MERATE, Seriate, Italy). TBE was performed with the patient in the upright position after fasting for at least 12 h before the examination. Patients ingested 150–200 mL of a low-density barium sulfate suspension, 45% weight/volume, over a short standardized interval, in order to achieve adequate esophageal filling while minimizing regurgitation and aspiration risk, frequent in dysphagic patients. Spot radiographs were obtained in the left posterior oblique projection at 1, 2, and, when indicated, 5 min after ingestion, maintaining consistent patient positioning and source-to-image geometry whenever possible (Figure 2).

The timed sequence was discontinued when complete esophageal clearance was documented at an earlier time point, in order to avoid unnecessary radiation exposure.

Patients remained upright throughout the timed sequence. The left posterior oblique projection was preferred because it reduces superimposition of the distal esophagus over the spine and cardiac silhouette and improves visualization of the esophagogastric junction.

### 2.3. Radiologic Measurements and Interobserver Reliability

**Figure 3:**
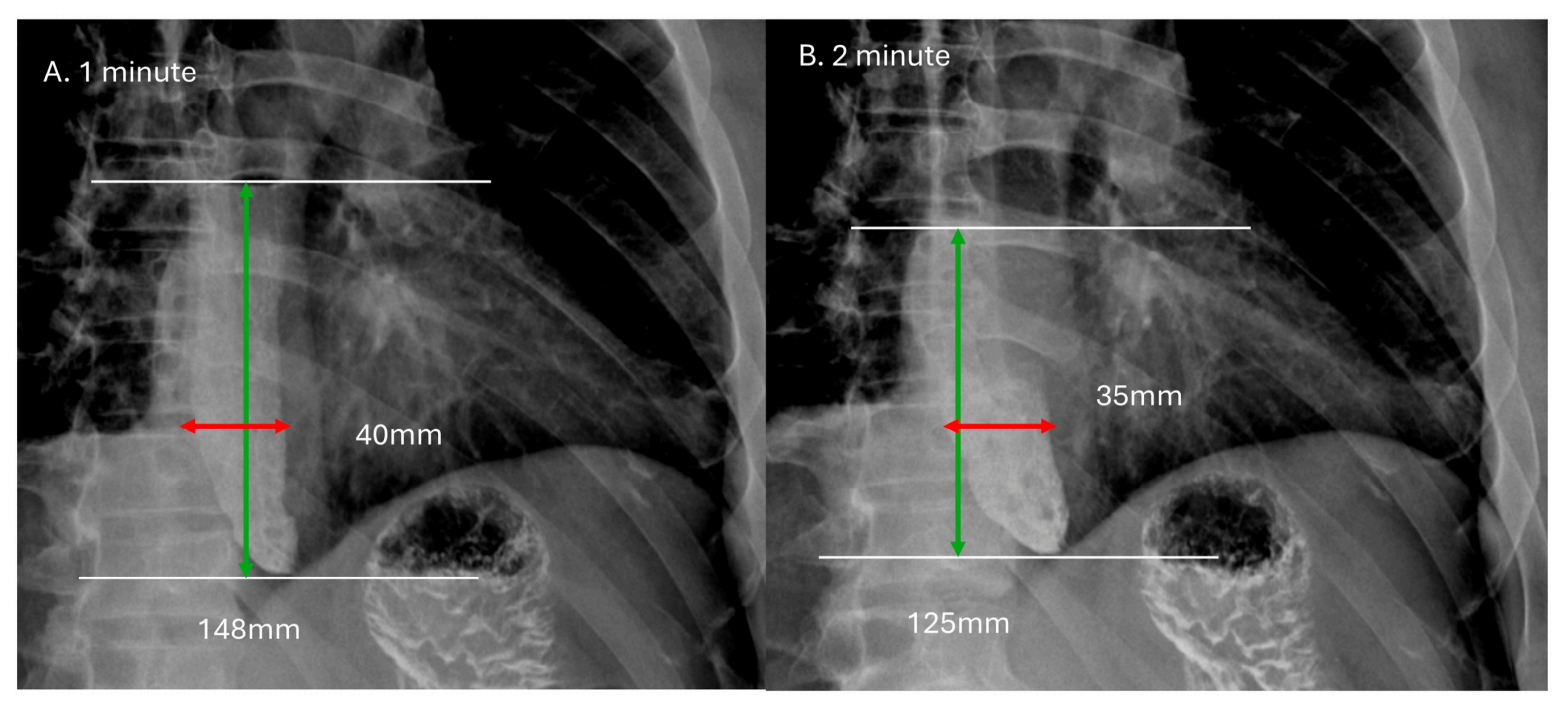
Representative timed barium esophagogram showing measurement of liquid barium retention over time. Left posterior oblique spot radiographs obtained at 1 min (**A**) and 2 min (**B**) after barium ingestion demonstrate persistent distal esophageal liquid barium retention. The vertical green line indicates barium column height, measured between the upper and lower limits of the retained contrast column, while the horizontal red line indicates maximal column width. In this example, column height decreased from 148 mm at 1 min to 125 mm at 2 min, and maximal width decreased from 40 mm to 35 mm.

Radiologic evaluation was performed by two radiologists who were blinded to HRM results: one senior radiologist with more than 20 years of experience in gastrointestinal functional gastrointestinal imaging and one radiologist with more than 7 years of experience. For each residual barium column, maximal width and craniocaudal height were measured in millimeters on Pictorial Archive and Communication System (PACS). Height was defined as the distance between the uppermost continuous barium level and the EGJ or distal point of hold-up. Width was measured perpendicular to the long axis of the esophagus at the widest reproducible point. The measurement approach is illustrated in Figure 3.

Following standardized TBE measurement principles, residual barium was quantified by measuring the height and width of a continuous intraluminal column on timed radiographs. For the purposes of the present radiologic analysis, a craniocaudal height of 2 mm was used as an operational minimum measurement criterion rather than as a clinically validated or disease-specific diagnostic cut-off. This threshold was intended to distinguish a continuous and reproducibly measurable residual barium column from minimal mucosal coating or small non-columnar traces of contrast. Accordingly, examinations showing a continuous residual esophageal barium column of at least 2 mm were classified as TBE-positive for the primary radiologic analysis. Minimal mucosal coating, small non-columnar traces of contrast, or residual contrast confined to a hiatal hernia were recorded as qualitative findings but were not considered sufficient to define TBE positivity. In addition, quantitative column height was descriptively stratified according to previously reported centimeter-based thresholds, including >5 cm at 1 min and >2 cm at 5 min [18]. Both readers first performed independent measurements of barium column height and width. Interobserver reliability for quantitative measurements was assessed using the intraclass correlation coefficient. Discrepant measurements were subsequently reviewed jointly, and a final consensus value was recorded for each measurement. Consensus values were used for descriptive quantitative analysis and for radiologic phenotype assignment.

### 2.4. Qualitative Radiologic Assessment

Qualitative descriptors were extracted from radiology reports and image review and grouped into non-mutually exclusive descriptive categories. Abnormal esophageal motor activity was recorded as a composite category encompassing dyskinetic, tertiary, ineffective, or atonic patterns; these descriptors were grouped for descriptive radiologic analysis and were not considered interchangeable diagnostic entities. Radiologic phenotypes were defined as follows: normal emptying, when no measurable residual barium column was recorded; transient stasis, when a measurable barium column was present at an early time point but decreased substantially or cleared on subsequent acquisitions; persistent stasis, when a measurable liquid barium column remained visible at 5 min; achalasia-like obstruction, when marked retention was associated with absent or minimal clearance and typical distal tapering or other radiologic features suggestive of achalasia; and hypercontractile or corkscrew-like stasis, when residual barium retention was associated with diffuse or segmental hypercontractile morphology rather than isolated EGJ-level hold-up.

The definition of positivity was intentionally separated from qualitative morphologic assessment. A measurable retained esophageal column was distinguished from minimal mucosal coating, small non-columnar traces of contrast, or residual contrast confined to a hiatal hernia. When available, the temporal behavior of the retained column was considered more informative than a single static measurement. Progressive reduction between time points suggested partial clearance, whereas stable or minimally changing retention suggested more severe functional impairment.

### 2.5. Hrm-Adjudicated Diagnostic Classification and Exploratory Concordance Analysis

Final diagnostic categories were assigned according to Chicago Classification v4.0 criteria. Because timed barium esophagography may contribute supportive evidence to the definition of clinically relevant EGJOO within the Chicago Classification v4.0 framework, the comparison between TBE findings and HRM-adjudicated diagnostic categories was interpreted as an exploratory concordance analysis rather than as an independent diagnostic accuracy validation. The TBE comparison was not intended to identify or retrospectively adjudicate individual HRM technical artifacts, transient manometric abnormalities, or medication-related motility effects. These potential confounders were not analyzed as separate variables in the present radiology-centered retrospective dataset.

For this analysis, patients with confirmed EGJOO and those with a provisional/non-confirmed EGJOO classification were included in the two-by-two table, whereas patients with achalasia or other final diagnoses were described separately and excluded from the EGJOO-specific concordance analysis.

A concordant-positive result was defined as a positive TBE examination in a patient classified as confirmed EGJOO. A discordant-negative result was defined as a negative TBE examination in a patient classified as confirmed EGJOO. A discordant-positive result was defined as a positive TBE examination in a patient classified as non-confirmed EGJOO. A concordant-negative result was defined as a negative TBE examination in a patient classified as non-confirmed EGJOO.

### 2.6. Statistical Analysis

Continuous variables were summarized descriptively as mean ± standard deviation. No inferential parametric comparisons requiring an assumption of normality were performed. Categorical variables are reported as absolute numbers and percentages. As an additional descriptive analysis, barium column height was also stratified according to literature-based centimeter thresholds, using >5 cm at 1 min and >2 cm at 5 min. A two-by-two concordance table was constructed in the HRM-adjudicated EGJOO subset, including confirmed EGJOO and non-confirmed EGJOO cases. Because TBE may contribute supportive evidence to the Chicago Classification v4.0 definition of clinically relevant EGJOO, sensitivity, specificity, positive predictive value, and negative predictive value were considered exploratory concordance metrics rather than independent diagnostic accuracy estimates. Exact binomial 95% confidence intervals were calculated for these metrics.

Interobserver reliability for quantitative barium column measurements was assessed using the intraclass correlation coefficient. Mean absolute differences between readers were also calculated to quantify the magnitude of measurement variability in millimeters. All statistical analyses were performed using IBM SPSS Statistics, version 32.0 (IBM Corp., Armonk, NY, USA).

## 3. Results

### 3.1. Study Population and Hrm-Adjudicated Diagnostic Categories

The study cohort consisted of 42 patients referred for TBE because of suspected EGJOO. Mean age was 58.0 ± 13.7 years, with age data available for all patients. There were 26 female patients (61.9%) and 16 male patients (38.1%). Final HRM-adjudicated diagnostic categories were assigned according to Chicago Classification v4.0 criteria. Twelve patients were classified as confirmed EGJOO and 6 as non-confirmed EGJOO. These 18 patients constituted the EGJOO-focused subset used for the exploratory two-by-two concordance analysis. Five patients were classified as achalasia and were described separately. The remaining patients had other final diagnoses, including ineffective esophageal motility in 6 cases, hiatal hernia-related findings in 10 cases, and nutcracker/hypercontractile esophagus in 3 cases.

Overall, TBE was positive in 12/42 patients (28.6%) and negative in 30/42 patients (71.4%). TBE positivity was observed in 5/12 patients with confirmed EGJOO, 0/6 patients with non-confirmed EGJOO, 5/5 patients with achalasia, 0/6 patients with ineffective esophageal motility, 0/10 patients with hiatal hernia-related findings, and 2/3 patients with nutcracker/hypercontractile esophagus. Residual contrast confined to a hiatal hernia was recorded as a qualitative morphologic finding but was not considered sufficient to define TBE positivity (Table 1). 

**Table 1:** Study population, final diagnostic categories, and global TBE results.

Variable	Value
Patients	42
Age, years	58.0 ± 13.9, available in 41 patients
Female sex	26/42 (61.9%)
Male sex	16/42 (38.1%)
Confirmed EGJOO	12/42 (28.6%)
Non-confirmed EGJOO	6/42 (14.3%)
Achalasia	5/42 (11.9%)
Ineffective esophageal motility	6/42 (14.3%)
Hiatal hernia-related findings	10/42 (23.8%)
Nutcracker/hypercontractile esophagus	3/42 (7.1%)
TBE positive	12/42 (28.6%)
TBE negative	30/42 (71.4%)
Morphologic findings reported	23/42 (54.8%)

### 3.2. Quantitative Tbe Findings

**Figure 4:**
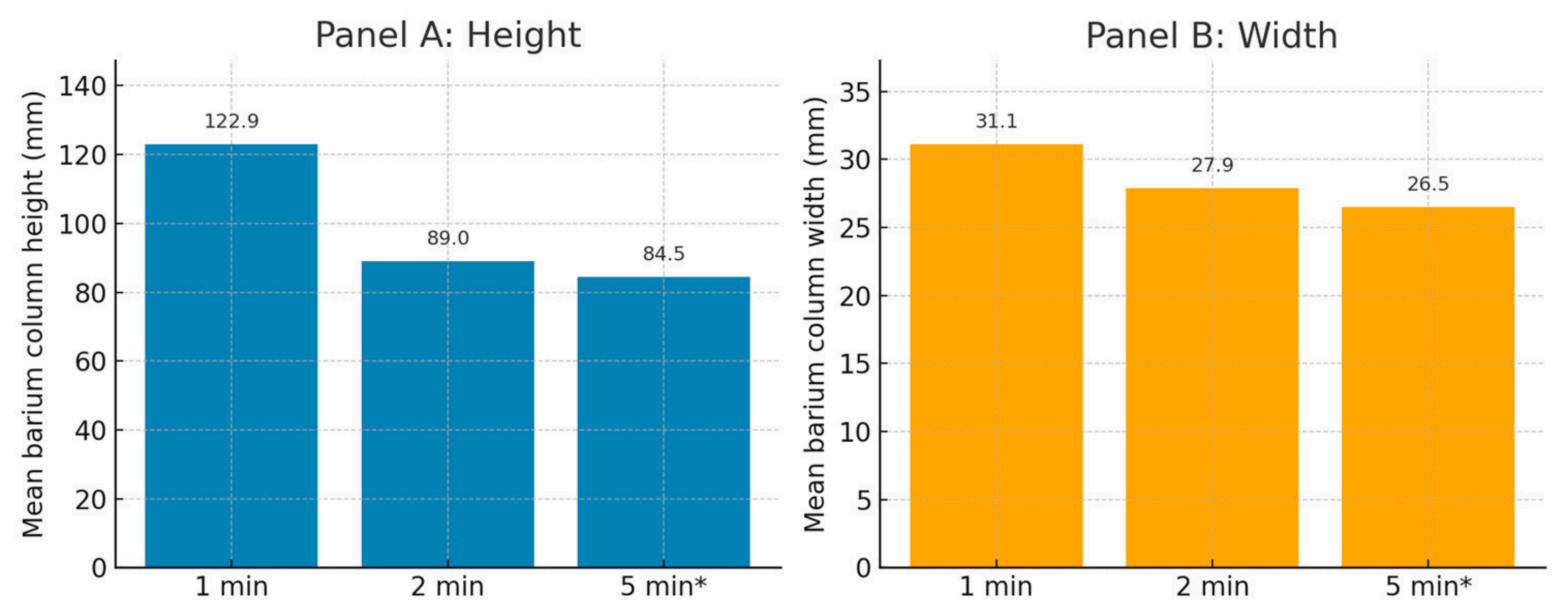
Distribution of quantitative barium column measurements over time in examinations with available measurements. Box plots show the median and interquartile range, with whiskers extending to 1.5 times the interquartile range. Individual data points are superimposed to display the complete distribution of observations. Panel (**A**) shows barium column height and Panel (**B**) maximal column width at 1, 2, and 5 min. Five-minute values include explicit zero values when complete clearance was documented (*).

Among TBE-positive examinations, measurable barium retention was available in all 12 patients at 1 min and 12 patients at 2 min. Mean barium column height decreased from 122.9 ± 65.4 mm at 1 min to 89.0 ± 54.6 mm at 2 min. Mean maximal width decreased from 31.1 ± 17.8 mm to 28.2 ± 18.2 mm over the same interval (Figure 4, Table 2). When barium column height was additionally stratified according to literature-based centimeter thresholds, 3/9 examinations (33.3%) showed residual retention > 5 cm at 1 min, whereas 6/9 (66.7%) showed a residual column < 2 cm. At 5 min, 1/9 examinations (11.1%) showed residual retention > 2 cm; this examination also showed a residual column > 5 cm. The remaining 8/9 examinations (88.9%) showed a residual column < 2 cm.

**Figure 5:**
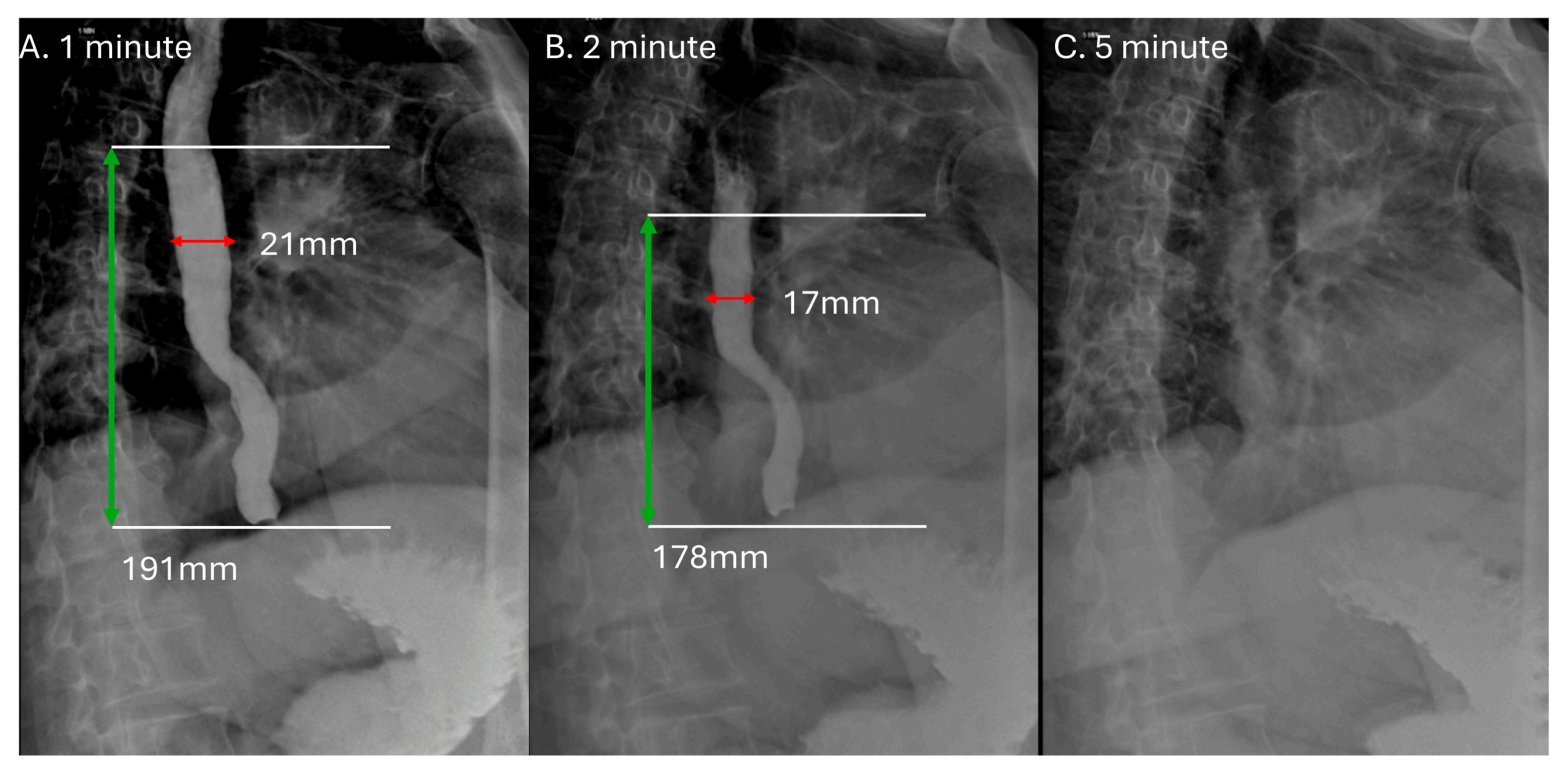
Representative timed barium esophagogram showing delayed clearance of liquid barium retention. Left posterior oblique radiographs obtained at 1 min (**A**), 2 min (**B**), and 5 min (**C**) after barium ingestion demonstrate progressive reduction and subsequent complete clearance of retained liquid barium. The vertical green line indicates barium column height, and the horizontal red line indicates maximal column width. In this example, column height decreased from 191 mm at 1 min to 178 mm at 2 min, while maximal width decreased from 21 mm to 17 mm. No residual liquid barium column was visible at 5 min.

**Figure 6:**
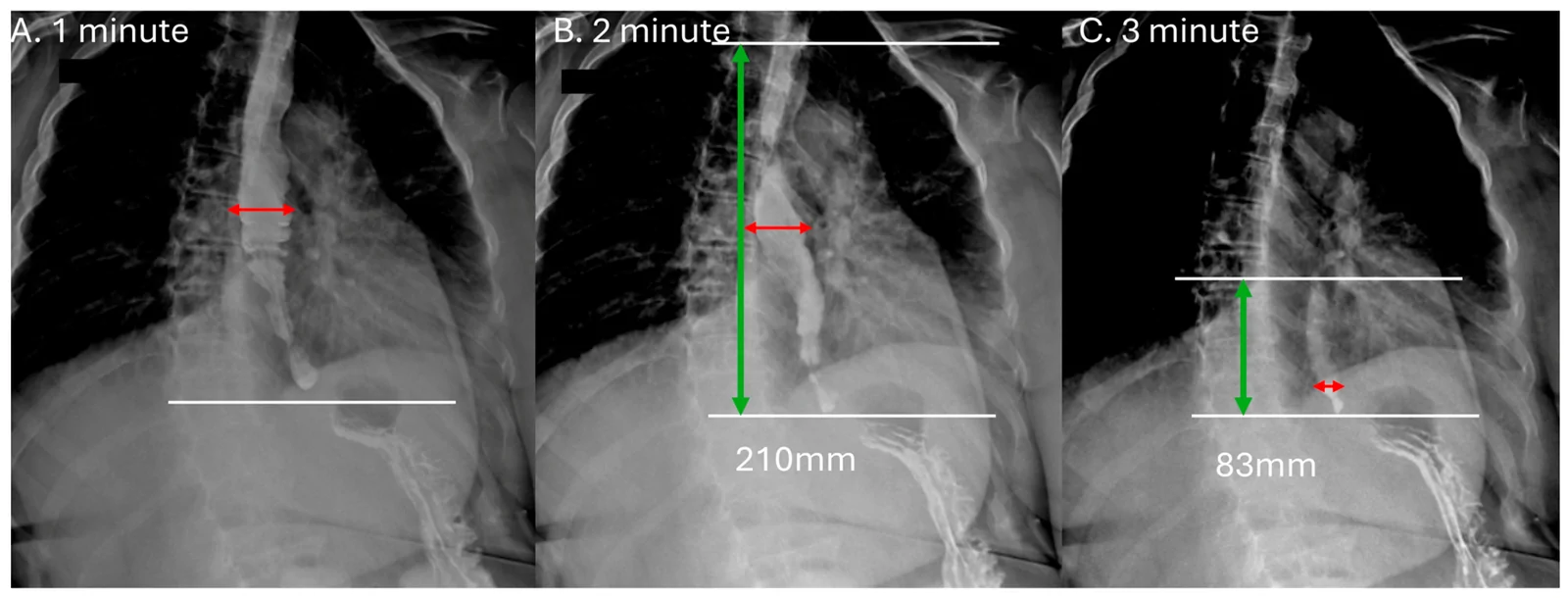
Representative timed barium esophagogram showing progressive reduction in retained barium. Left posterior oblique radiographs obtained at 1 min (**A**), 2 min (**B**), and 5 min (**C**) after barium ingestion demonstrate persistent but progressively decreasing esophageal barium retention. The vertical green line indicates barium column height, measured between the upper and lower limits of the retained contrast column, while the horizontal red arrow indicates maximal column width. In this example, barium column height decreased from 210 mm at 2 min to 83 mm at 5 min, indicating partial but incomplete clearance.

Five-minute measurements were available in 9 examinations, including examinations in which delayed imaging showed complete clearance after earlier retention. Mean 5 min height was 42.7 ± 68.8 mm and mean width was 17.3 ± 30.0 mm when explicit zero values were included. Persistent retention greater than zero at 5 min was present in two patients; among these persistent-stasis cases, mean residual height was 128.0 ± 42.4 mm and mean residual width was 52.0 ± 29.7 mm. Representative examples of delayed complete clearance and partial but incomplete clearance are shown in Figure 5 and Figure 6, respectively.

### 3.3. Interobserver Reliability

Interobserver reliability for quantitative barium column measurements was excellent, with an intraclass correlation coefficient of 0.90 and a mean absolute inter-reader difference of <3 mm (Supplementary Table S1). Discrepancies were resolved by joint review, and final consensus measurements were used for the quantitative analysis. The limited magnitude of disagreement suggests that variability was mainly related to minor technical differences in the placement of height and width axes rather than to disagreement in the identification of residual barium retention.

### 3.4. Radiologic Phenotypes and Morphologic Findings

Normal or rapidly clearing examinations accounted for 30/42 studies (71.4%). The remaining 12 examinations were positive and included cases with transient liquid stasis, persistent stasis, achalasia-like obstruction, and hypercontractile or corkscrew-like stasis.

**Figure 7:**
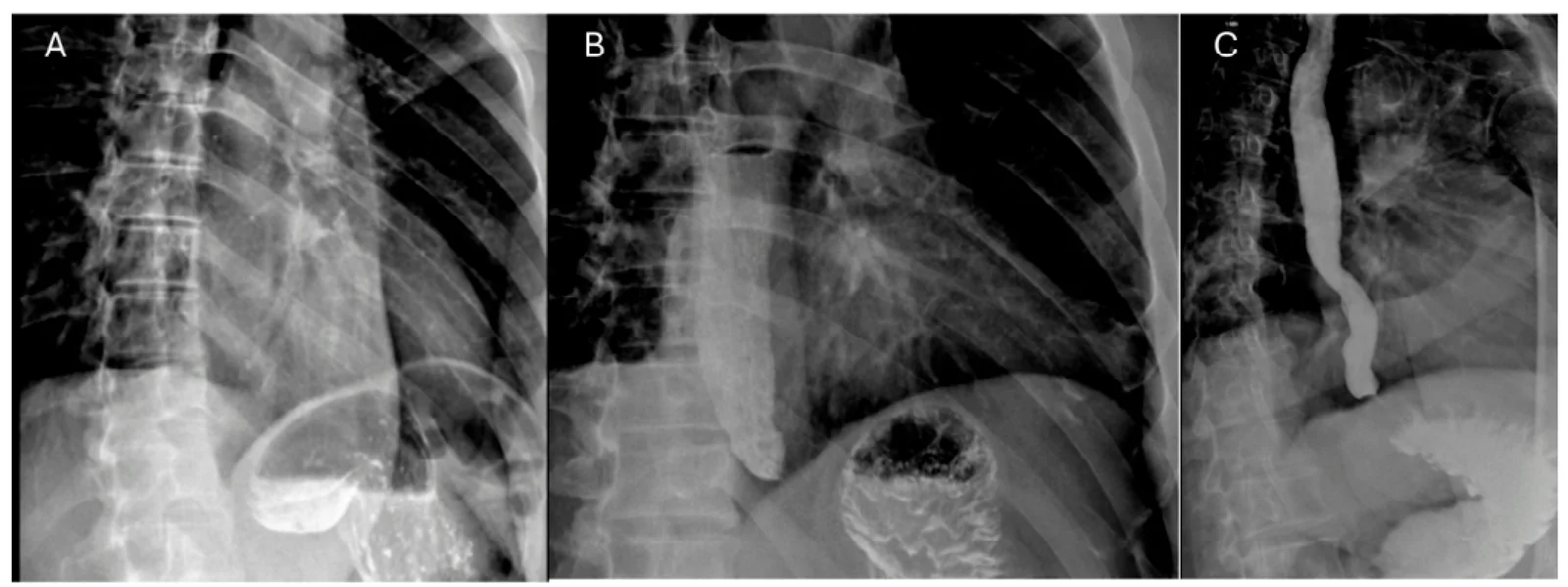
Spectrum of timed barium esophagogram appearances. Representative left posterior oblique radiographs showing different TBE patterns: normal or near-complete esophageal emptying (**A**), distal esophageal/EGJ-level contrast retention with marked barium stasis and achalasia-like distal tapering (**B**), and corkscrew-like esophageal morphology characterized by segmental narrowing and alternating abnormal contractions producing a spastic, non-linear luminal contour, associated with segmental contrast stasis (**C**).

Qualitative morphologic abnormalities were recorded in 23/42 radiologic examinations and were particularly frequent among TBE-positive studies (Table 3). These abnormalities included dyskinetic or tertiary contractions, mucosal irregularity or esophagitis, hiatal hernia, esophageal dilation or megaesophagus, distal tapering or abnormal EGJ morphology, diverticular findings, air-fluid level, and corkscrew-like morphology. Representative examples of the principal radiologic phenotypes are shown in Figure 7.

The distribution of qualitative findings highlights the added value of image-based interpretation. TBE-positive studies were not characterized only by numeric retention, but also by visible motor or structural abnormalities. Abnormal esophageal motor patterns represented the most frequent qualitative category, supporting the concept that impaired emptying may coexist with a variety of non-propulsive or abnormal motor patterns. Dilation, abnormal distal narrowing, corkscrew-like morphology, and air-fluid level were less frequent but more specific-appearing radiographic signs of advanced functional impairment or overlap with achalasia-like or hypercontractile physiology.

Conversely, several TBE-negative examinations contained minor qualitative findings, including reflux, hiatal hernia, or mucosal irregularity. These findings were not interpreted as evidence of EGJ outflow obstruction in the absence of a measurable retained esophageal column. This separation between obstruction-related functional signs and incidental or associated morphologic findings is important for reporting, because it prevents overcalling EGJOO on the basis of non-specific abnormalities that may be common in patients referred for dysphagia.

**Table 3:** Qualitative radiologic findings extracted from TBE reports and image review. Categories are non-mutually exclusive.

Qualitative Radiologic Finding	All Patients, *n*/42
Abnormal esophageal motor pattern (dyskinetic, tertiary, ineffective, or atonic activity)	12
Reflux or cardial incompetence	4
Esophagitis, mucosal irregularity, or fold thickening	7
Hiatal or sliding hernia	5
Esophageal dilation, megaesophagus, or dolichomegaesophagus	3
Distal tapering, distal narrowing, or abnormal EGJ morphology	5
Diverticulum or pseudodiverticula	2
Air-fluid level	1
Corkscrew-like morphology	2

### 3.5. Distribution of Tbe Results Across Diagnostic Categories

The distribution of TBE results across final HRM-adjudicated diagnostic categories is shown in Table 4. Among patients with diagnoses other than confirmed EGJOO, TBE positivity was observed in 5/5 achalasia cases and 2/3 patients with nutcracker/hypercontractile esophagus. No patient with non-confirmed EGJOO or ineffective esophageal motility had a positive TBE. Hiatal hernia-related findings were not displayed as a separate category in Table 4 because residual contrast confined to a hiatal hernia was considered a qualitative finding and did not meet the predefined criteria for TBE positivity.

In the nutcracker/hypercontractile subgroup, barium retention was interpreted as diffuse or segmental stasis related to hypercontractile activity and corkscrew-like morphology rather than as isolated EGJ-level hold-up. This finding supports the role of TBE as a functional and morphologic examination capable of identifying impaired bolus transit across different esophageal motor patterns, rather than as a disease-specific test for EGJOO alone.

### 3.6. Exploratory Concordance Analysis in the Egjoo-Focused Subset

The EGJOO-focused subset included 18 patients: 12 with confirmed EGJOO and 6 with non-confirmed EGJOO. In this subset, the two-by-two concordance table included 5 concordant-positive, 7 discordant-negative, 6 concordant-negative, and 0 discordant-positive TBE examinations (Table 5). Exploratory concordance metrics were therefore 41.7% for sensitivity-like concordance, 100% for specificity-like concordance, 100% for positive predictive concordance, and 46.2% for negative predictive concordance (Table 6).

These metrics were interpreted as exploratory rather than as independent diagnostic accuracy estimates, because TBE may contribute supportive evidence to the Chicago Classification v4.0 definition of clinically relevant EGJOO.

**Table 5:** Two-by-two exploratory concordance table for TBE in the EGJOO-focused subset.

	Confirmed EGJOO	Non-Confirmed EGJOO
TBE positive	5	0
TBE negative	7	6

**Table 6:** Exploratory concordance metrics for TBE in the EGJOO-focused subset.

Metric	Value	95% CI
Sensitivity-like concordance	41.7%	15.2–72.3%
Specificity-like concordance	100%	54.1–100%
Positive predictive concordance	100%	47.8–100%
Negative predictive concordance	46.2%	19.2–74.9%

## 4. Discussion

This retrospective radiologic assessment supports the role of standardized TBE as a functional and morphologic examination in patients referred for suspected EGJOO. The main finding is that radiologically detectable residual barium retention, defined for the primary analysis using an operational minimum measurable column height of 2 mm, provided visible evidence of incomplete bolus clearance and showed high exploratory concordance with clinically relevant EGJOO according to Chicago Classification v4.0. Importantly, this 2 mm criterion should not be interpreted as a clinically validated diagnostic threshold. Conversely, a negative TBE did not exclude confirmed EGJOO, confirming that TBE should be interpreted as a complementary test rather than as a standalone diagnostic gatekeeper.

The EGJOO-focused subset showed 5 concordant-positive, 7 discordant-negative, 6 concordant-negative, and no discordant-positive examinations. These results translated into high specificity-like and positive predictive concordance, but limited sensitivity-like and negative predictive concordance. This pattern is clinically meaningful. When residual barium retention is demonstrated above the EGJ, particularly in association with compatible morphology and delayed clearance, the radiologist provides objective evidence that the manometric abnormality has a functional consequence [31]. However, the absence of retention during the examination does not rule out EGJOO, especially when obstruction is mild, intermittent, or partially compensated by preserved peristaltic function [2,3,5,9].

These concordance metrics require cautious interpretation. Because TBE may serve as supportive evidence within the Chicago Classification v4.0 framework for clinically relevant EGJOO, the present analysis should not be considered an independent diagnostic accuracy validation against a fully separate reference standard. Rather, it describes the relationship between radiologic evidence of impaired bolus transit and the final HRM-adjudicated clinical classification. This limitation may partly increase apparent specificity-like and positive predictive concordance, and future studies should ideally include an adjudication process that does not incorporate the TBE result.

The limited sensitivity-like concordance observed in this cohort should not be interpreted as failure of the radiologic test. HRM and TBE measure different aspects of esophageal physiology [32,33]. HRM identifies pressure patterns during controlled swallows, whereas TBE evaluates the macroscopic result of bolus transport during upright imaging. A patient may meet Chicago Classification v4.0 criteria for EGJOO and still clear a liquid barium bolus rapidly, particularly when peristaltic reserve is preserved [6,26,29,33,34]. In this setting, a negative TBE indicates that functionally relevant radiographic retention was not demonstrated under the conditions of the examination, not that the manometric diagnosis is necessarily incorrect.

The temporal dimension of TBE is particularly important [16,35]. Early retention at 1 min indicates delayed emptying, but does not by itself define severity. Previous studies have proposed substantially higher centimeter-based thresholds for clinically relevant esophageal stasis, particularly a barium column height > 5 cm at 1 min and >2 cm at 5 min. In the present cohort, 3/9 examinations exceeded 5 cm at 1 min, whereas only 1/9 exceeded 2 cm at 5 min, indicating that marked persistent stasis was uncommon despite the presence of measurable early retention. Progressive reduction between time points suggests partial or compensated clearance, whereas persistent retention at 5 min identifies a more severe emptying phenotype. In the present cohort, persistent retention at 5 min was uncommon and was documented in two patients. This finding suggests that delayed 5 min stasis may represent a more advanced radiologic phenotype, while transient early stasis may reflect milder or partially compensated obstruction.

The morphologic component of TBE also contributed substantially to interpretation. Qualitative abnormalities were frequent among TBE-positive examinations and included dyskinetic or tertiary contractions, mucosal irregularity or esophagitis, esophageal dilation or megaesophagus, abnormal distal EGJ morphology, diverticular findings, air-fluid level, and corkscrew-like morphology. These findings are important because they help distinguish isolated EGJ-level hold-up from broader motor or structural patterns. For this reason, TBE should not be reduced to a binary positive or negative result. The spatial distribution of retention, the clearance trend, and the associated morphology should all be incorporated into the final radiologic impression.

The finding of TBE positivity in all patients with achalasia was expected and reinforces the ability of TBE to depict severe impairment of esophageal emptying. More interestingly, TBE positivity was also observed in patients with nutcracker or hypercontractile esophagus. In these cases, retention was interpreted as diffuse or segmental stasis related to hypercontractile activity and corkscrew-like morphology rather than isolated EGJ-level hold-up. Conversely, one patient with a nutcracker/hypercontractile manometric pattern had a negative TBE, with no measurable residual esophageal barium column. This finding illustrates that increased contractile vigor does not necessarily translate into impaired macroscopic bolus clearance. Preserved propulsive function or the intermittent expression of the motor abnormality may allow for effective esophageal emptying despite an abnormal HRM pattern. These observations emphasize that barium retention is not disease-specific for EGJOO and that the functional consequences of hypercontractile motility may be heterogeneous. Instead, TBE functions as a radiologic test of bolus transit that must be interpreted within the complete imaging pattern and clinical–manometric context.

The distinction between true esophageal retention and non-obstructive residual contrast is another practical point. In this study, residual contrast confined to a hiatal hernia was recorded as a qualitative finding but was not considered sufficient to define TBE positivity. This approach avoids overcalling impaired esophageal emptying in patients whose contrast residue is related to an anatomic pouch rather than to a retained esophageal column. Similarly, minimal mucosal coating or small non-columnar traces of contrast should be separated from a measurable continuous barium column. These distinctions are essential for reproducible reporting and for maintaining the clinical specificity of the examination.

Interobserver reliability for quantitative barium column measurements was excellent, with an ICC of 0.90 and a mean absolute difference between readers of less than 3 mm. This supports the reproducibility of PACS-based height and width measurements when a standardized measurement method is used. Minor differences between readers likely reflected technical variability in the placement of height and width axes rather than disagreement in recognizing residual barium retention. Final consensus measurements were used for analysis, but the high ICC suggests that quantitative TBE parameters can be reliably incorporated into structured radiologic reporting.

From a practical radiology perspective, these results support a structured TBE report in suspected EGJOO. The report should include technical adequacy, bolus type and volume, time points acquired, whether delayed imaging was omitted after complete clearance, barium column height and width when measurable, clearance trend over time, EGJ morphology, esophageal morphology, visible motor pattern, and a final functional interpretation. Such a structure may improve communication with gastroenterologists and help integrate TBE findings into multidisciplinary decision-making.

Clinically, a positive TBE may increase confidence that symptoms and manometric findings are associated with impaired bolus transit. This may support closer clinical correlation, further evaluation, or treatment planning in selected patients. A negative TBE, however, should be interpreted cautiously and should not automatically dismiss symptoms or HRM abnormalities. The test is best positioned as part of a diagnostic pathway that integrates symptoms, endoscopy, HRM, supportive testing, and clinical evolution.

Overall, the present study reinforces the radiologic value of TBE in suspected EGJOO while also defining its limitations. A positive examination provides visible and measurable evidence of impaired esophageal emptying, but the meaning of that retention depends on its location, persistence, and morphology. A negative examination does not exclude clinically relevant EGJOO. Future prospective studies should validate standardized quantitative thresholds, collect dose metrics, assess reader agreement for both quantitative and qualitative features, and evaluate whether radiologic phenotypes predict symptoms, treatment selection, and post-treatment outcomes.

## 5. Strength and Limitations

The main strength of this study is that it presents a radiology-centered assessment of esophageal emptying in a clearly defined cohort of patients referred for suspected EGJOO. The manuscript integrates quantitative barium column measurements, temporal clearance patterns, qualitative morphologic interpretation, and interobserver reliability assessment. This approach reflects the practical value of TBE as a functional and morphologic examination rather than as a purely binary test.

Another strength is the explicit separation between measurable esophageal barium retention and non-obstructive residual contrast. By requiring a continuous residual esophageal barium column with a craniocaudal height of at least 2 mm to define TBE positivity, and by excluding minimal mucosal coating or contrast confined to a hiatal hernia, the study applies a practical radiologic definition aimed at preserving specificity and reducing overinterpretation of minor findings.

### Limitations

Several limitations should be acknowledged. First, the cohort was relatively small, particularly the EGJOO-focused subset used for the exploratory concordance analysis, resulting in wide confidence intervals and requiring the findings to be regarded as hypothesis-generating. Moreover, because TBE may itself contribute supportive evidence within the Chicago Classification v4.0 framework for clinically relevant EGJOO, the concordance analysis cannot be interpreted as an independent diagnostic accuracy validation against a fully separate reference standard; future studies should therefore use an adjudication process independent of TBE findings when diagnostic performance is being evaluated. Although interobserver reliability for quantitative barium column measurements was excellent (ICC 0.90), final measurements were obtained after consensus review, and formal interobserver agreement was not separately assessed for qualitative morphologic descriptors or radiologic phenotype assignment. In addition, qualitative morphologic categories were partly derived from radiology reports and retrospective image review and should therefore be regarded as descriptive rather than prospectively standardized variables. Radiation exposure metrics, including dose-area product, were not available in the final dataset, precluding formal dose assessment and comparison with alternative fluoroscopic or radiographic protocols. Finally, longitudinal clinical follow-up was not available; consequently, the prognostic value of TBE phenotypes for symptom evolution, treatment selection, and post-treatment response could not be assessed. Prospective studies with standardized radiologic descriptors, independent diagnostic adjudication, radiation dose recording, formal qualitative interobserver assessment, and longitudinal clinical follow-up are warranted to address these limitations. In addition, external validity may be influenced by the single-center setting, the use of a specific radiographic system and acquisition protocol, and the dependence of TBE measurements on standardized contrast administration, patient positioning, image acquisition, and reader experience; therefore, direct generalization to centers using different equipment or technical protocols should be made cautiously.

Several limitations should be acknowledged. First, the cohort is relatively small, particularly the EGJOO-focused subset used for exploratory concordance analysis. The confidence intervals around concordance metrics are therefore wide, and the results should be considered hypothesis-generating. Second, because TBE may contribute supportive evidence to the Chicago Classification v4.0 definition of clinically relevant EGJOO, the concordance analysis should not be interpreted as an independent diagnostic accuracy validation. Future studies should include an adjudication process independent of TBE findings when the objective is to validate diagnostic performance.

Third, although interobserver reliability for quantitative barium column measurements was excellent, with an ICC of 0.90, final values were obtained after consensus review. Therefore, the present analysis supports the reproducibility of quantitative measurements but does not separately assess interobserver agreement for qualitative morphologic descriptors or radiologic phenotype assignment. Future prospective studies should evaluate both quantitative and categorical agreement using predefined structured reporting forms.

Fourth, qualitative morphologic categories were partly extracted from radiology reports and retrospective image review. Although these descriptors reflect clinically meaningful imaging features, they should be regarded as descriptive rather than prospectively standardized variables. A dedicated structured TBE reporting template would improve future data collection and facilitate reproducibility across readers and centers.

Fifth, radiation exposure metrics such as dose-area product were not available in the final dataset. This prevents formal dose reporting and limits comparison with other fluoroscopic or radiographic protocols. Nevertheless, the standardized timed protocol, including omission of delayed imaging after complete early clearance, is consistent with a radioprotection-oriented approach. Future studies should include dose-area product, number of images, and whether the 5 min acquisition was acquired or omitted, so that diagnostic yield can be balanced against radiation exposure.

Finally, the study lacks longitudinal clinical follow-up. Therefore, the prognostic value of TBE phenotypes for symptom evolution, treatment selection, or post-treatment response could not be assessed. Longitudinal studies are needed to determine whether baseline retention pattern, persistence at 5 min, or associated morphology can predict clinical outcomes in patients with suspected or confirmed EGJOO.

## 6. Clinical Implications

In clinical practice, a positive TBE provides direct radiologic evidence of impaired bolus transit and may support further evaluation or treatment planning in symptomatic patients with compatible HRM findings. A negative TBE should be interpreted more cautiously. It may indicate absent radiologic obstruction at the time of examination, but it does not exclude clinically relevant EGJOO. The examination is therefore best positioned as a complementary functional and morphologic imaging test within a multidisciplinary diagnostic pathway.

For radiology departments, these findings support the implementation of a standardized TBE pathway. The examination does not require advanced post-processing, but it does require consistent timing, reproducible positioning, defined measurement rules, and a report that separates true esophageal retention from associated anatomic or mucosal findings. This makes TBE particularly suitable for centers evaluating esophageal dysphagia and suspected motor disorders, provided that acquisition and reporting are standardized.

For clinicians, the most actionable message is that a positive TBE should increase confidence that symptoms and HRM findings are associated with impaired bolus transit. Conversely, a negative TBE should prompt careful reassessment rather than automatic dismissal of symptoms or manometric abnormalities. Imaging results should be integrated with dysphagia severity, endoscopic findings, HRM metrics, supportive testing, and clinical evolution. In this framework, radiology contributes a visible functional endpoint that can support patient selection, treatment planning, and follow-up assessment.

### 6.1. Structured Radiologic Reporting Proposal

Based on the present findings, TBE reporting in suspected EGJOO should include both quantitative and qualitative components (Table 7). A structured report should document technical adequacy, contrast type and volume, time points acquired, reason for omission of delayed imaging when applicable, barium column height and width when measurable, clearance trend over time, distal esophageal and EGJ morphology, visible motor pattern, associated findings, and a final functional interpretation.

**Table 7:** Suggested structured reporting items for TBE in suspected EGJOO.

Reporting Item	Recommended Content
Technical adequacy	Patient position, contrast type and volume, time points acquired, and any omitted acquisition.
Quantitative emptying	Column height and maximal width at 1, 2, and 5 min when measurable.
Operational measurement criterion	Continuous residual esophageal barium column with craniocaudal height ≥ 2 mm; used as a minimum measurable threshold rather than a clinically validated diagnostic cut-off.
Clinically oriented quantitative interpretation	Consider reporting centimeter-based retention thresholds, including >5 cm at 1 min and >2 cm at 5 min, together with continuous column measurements
Clearance pattern	Normal emptying, transient stasis, persistent stasis, achalasia-like obstruction, or hypercontractile/corkscrew-like stasis.
EGJ morphology	Smooth tapering, distal narrowing, bird-beak-like appearance, cardial opening, or post-treatment change.
Esophageal morphology	Dilation, tortuosity, sigmoidization, diverticula, mucosal irregularity, hiatal hernia, or air-fluid level.
Motility impression	Visible tertiary, dyskinetic, ineffective, hypercontractile, or non-propulsive contractions.
Associated non-obstructive findings	Mucosal coating, reflux, hiatal hernia, or residual contrast confined to a hernia, when present.
Final impression	Radiologic evidence present or absent for impaired bolus transit, with phenotype and differential considerations.

### 6.2. Future Directions

Future research should move from retrospective extraction of TBE variables to prospective structured radiologic data collection. A dedicated TBE worksheet could capture ingestion adequacy, time points acquired, height and width for each reader, morphology, phenotype, and dose metrics in a standardized format. Such a workflow would allow robust evaluation of reproducibility, automated calculation of clearance percentages, and multicenter comparison of TBE findings.

Longitudinal follow-up is another important step. In achalasia, persistence of barium retention after therapy is clinically meaningful; whether similar radiologic thresholds can predict outcome in EGJOO remains uncertain. A prospective study linking baseline TBE phenotype to symptom evolution, need for therapy, and post-treatment clearance would help determine whether TBE is only a diagnostic support test or also a prognostic imaging biomarker.

Future studies should also evaluate whether a combination of quantitative thresholds, persistence at 5 min, and qualitative morphology improves patient stratification. In particular, distinguishing transient EGJ-level stasis from persistent stasis, achalasia-like obstruction, and hypercontractile/corkscrew-like stasis may help refine the role of radiology in the multidisciplinary evaluation of esophageal motor disorders.

## 7. Conclusions

In this patient cohort standardized TBE was positive in approximately one third of examinations. In the HRM-adjudicated EGJOO subset, positive TBE findings showed favorable specificity-like and positive predictive concordance point estimates with clinically relevant EGJOO according to Chicago Classification v4.0; however, the wide confidence intervals and small sample size require cautious interpretation. Conversely, a negative TBE did not exclude confirmed EGJOO. The radiologic value of TBE extends beyond binary positivity. Systematic assessment of temporal clearance and esophageal morphology allows for classification into clinically useful phenotypes, including transient stasis, persistent stasis, achalasia-like obstruction, and hypercontractile/corkscrew-like stasis. Future studies should prospectively standardize radiologic descriptors, include independent adjudication not incorporating TBE results, collect dose metrics, and evaluate whether TBE phenotypes predict treatment response.

## Figures and Tables

**Table 2:** Quantitative barium column measurements in TBE-positive examinations.

TBE Phase and Time Point	Column Height (mm)
1 min	122.9 ± 65.4
2 min	89.0 ± 54.6
5 min, including explicit zero values	42.7 ± 68.8
5 min, residual retention only	128.0 ± 42.4

**Table 4:** Distribution of TBE results across final HRM-adjudicated diagnostic categories.

Diagnostic Category	*n*	TBE Positive	TBE Negative
Confirmed EGJOO	12	5	7
Non-confirmed EGJOO	6	0	6
Achalasia	5	5	0
Ineffective esophageal motility	6	0	6
Nutcracker/hypercontractile esophagus	3	2	1
Total	42	12	30

## Data Availability

Data are available upon request to the corresponding author due to privacy restrictions.

## Supplementary Materials

The following supporting information can be downloaded at https://www.mdpi.com/article/10.3390/diagnostics16172743/s1, Table S1: Summary of interobserver reliability for quantitative TBE measurements.
