# Supplementary material for: Timed Barium Esophagography in Esophagogastric Junction Outflow Obstruction: A Retrospective Radiologic Assessment
Source: Diagnostics (Basel). 2026 Aug 27;16(17):2743. doi: 10.3390/diagnostics16172743 (PMC13565209; doi:10.3390/diagnostics16172743)
Supplement: Supplementary file 1 [file diagnostics-16-02743-s001.zip › diagnostics-4465656-supplementary.pdf]

Supplementary Table S1. Summary of interobserver reliability for quantitative TBE measurements

Reliability parameter	Result	Interpretation
Overall intraclass correlation coefficient (ICC)	0.90	Excellent interobserver reliability
Mean absolute inter-reader difference	<3 mm	Limited measurement variability
Final measurements used for quantitative analysis	Consensus values	Obtained after independent reader assessment and joint review

Footnote: *ICC, intraclass correlation coefficient. Quantitative barium column height and width measurements were initially performed independently by the two radiologists. Discrepant measurements were subsequently reviewed jointly, and consensus values were used for the final quantitative analysis.*
